# Tradition, modernity, and innovation in maritime balneology

**Published:** 2016

**Authors:** VL Purcarea

**Affiliations:** * “Carol Davila” University of Medicine and Pharmacy, Bucharest, Romania

During 1-4th September 2016, “Maritime Balneology, Physical and Rehabilitation Medicine – Tradition, Modernity and Innovation” National Conference with international participation, took place in Techirghiol under the aegis of the International Society of Medical Hydrology and Climatology (ISMHC) and the International Spinal Cord Society (ISCoS), the event celebrating the International Spinal Cord Injury (SCI) – Awareness – Day.

The Conference also celebrated 117 years of Balneology in Techirghiol, and was organized by Techirghiol Spa and Rehabilitation Sanatorium (TSRS) together with the Balkan Environmental Association (B.EN.A.), Romanian Society of Spinal Cord Pathology, Therapy and Rehabilitation (RoSCoS), “Carol Davila” University of Medicine and Pharmacy, Bucharest, Romanian Balneology Association (RBA), “Ovidius” University in Constanta, National Institute of Rehabilitation, Physical Medicine and Balneoclimatology (NIRPMB), Romanian Society of NeuroRehabilitation (RoSNeRa), “Grigore Antipa” National Institute for Marine Research and Development in Constanta. 

**Fig. 1:**
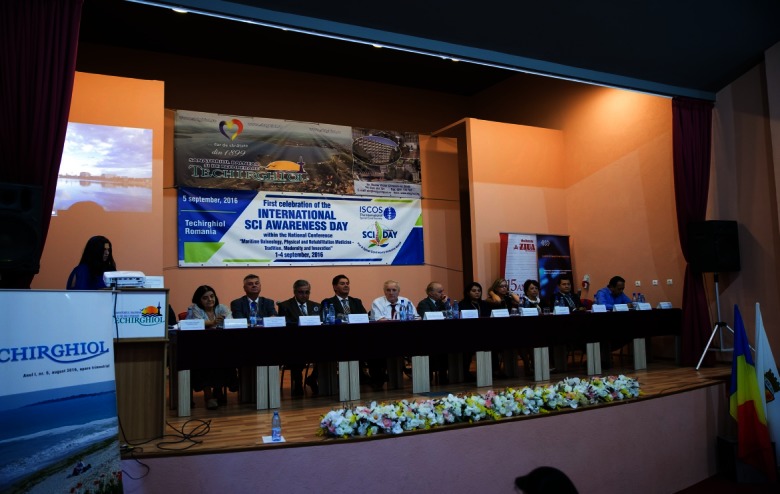
The opening ceremony

The opening ceremony included the welcome notes of the members of the presidium, who were also evidenced in the picture above: from left to right, Lawyer Elena-Roxana Almăşan, PhD student and also manager of Techirghiol Spa and Rehabilitation Sanatorium (TSRS) - President of the Conference Organizing Committee; Iulian Soceanu, Mayor of Techirghiol; Prof. Adriana S. Nica, MD, PhD, - “Carol Davila” University of Medicine and Pharmacy, Bucharest and National Institute of Rehabilitation, Physical Medicine and Balneoclimatology (NIRPMB), Bucharest; Prof. Sorin Rugină, PhD, Rector of “Ovidius” University in Constanta - Honorary President of the Conference; Prof. Victor Lorin Purcărea, PhD, “Carol Davila” University of Medicine and Pharmacy in Bucharest - President of the Conference; Prof. Gelu Onose, MD, PhD, “Carol Davila” University of Medicine and Pharmacy, Bucharest and “Bagdasar-Arseni” Clinical Emergency Hospital in Bucharest - President of the Conference and Executive Honorary President of the Romanian Society of Rehabilitation, Physical Medicine and Balneoclimatology (RSRPMB); Acad. Prof. Marian Traian Gomoiu, MD, PhD, Constanța, Corresponding member of the Romanian Academy, Acad. Prof. Constantin Ionescu-Tîrgoviște, MD, PhD, Bucharest – Honorary President of the Conference; Mariana Golumbeanu, PhD, Senior Researcher II, Constanţa – Balkan Environmental Association (B.EN.A.) and Vice-president of the Scientific Committee of the Conference; Assoc. Prof. Zacharoula Andreopoulou, Greece – “Aristotle” University in Thessaloniki and B.EN.A.; Tania Zaharia, PhD, Senior Researcher I – “Grigore Antipa” National Institute for Marine Research and Development in Constanta; Biol. Constantin Munteanu, MD, PhD – President of the Romanian Association of Balneology (RAB) and Vice-president of the Conference Organizing Committee; Lecturer Dan Blendea, MD, PhD – President of RSRPMB and of Specialty Advisory Commission of the Ministry of Health and Vice-president of the Scientific Committee of the Conference.

The main objectives of the event were to stimulate the interest of the scientific community regarding the acknowledgement and valorization of the sludge in Lake Techirghiol as a sanogeneous and therapeutical-rehabilitative factor and to impel the continuation and development of the research demarches in this field and respectively of celebrating the International Spinal Cord Injury (SCI) – Awareness – Day, which has been recently established by the International Spinal Cord Society (ISCoS) together with the World Health Organization (WHO), as both an expression of the new, leading trend in physical medicine and rehabilitation, meaning the one of neurorehabilitation, which represents a field in which TSRS is also known as an extremely competent and experienced medical unit. 

**Fig. 2:**
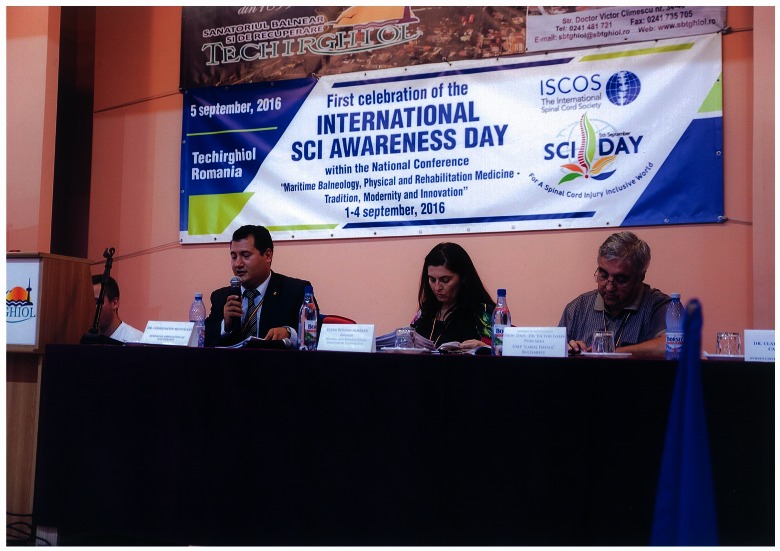
Presidium of the “Maritime Balneology, Physical and Rehabilitation Medicine – Tradition, Modernity and Innovation” National Conference

The celebration of the International Spinal Cord Injury (SCI) – Awareness – Day was possible for the first time in Romania, one of the first countries that adhered to this initiative of ISCoS and WHO, thanks to a tight collaboration between RoSCoS (affiliated to ISCoS) and TSRS, by organizing a dedicated session of scientific presentations during the Conference in Techirghiol. 

 The themes approached in the Conference were Balneology; Physical Medicine and Rehabilitation; Integrated Balneary Management, Environment Protection, Resources and Legislation; Kinesiotherapy in Balneology and Rehabilitation; Spinal Cord Pathology, Therapy and Rehabilitation; and many others. 

The participants in the event considered the subjects presented interesting, useful, and new.

Among the works presented, the following should be mentioned: Integrated management of patients in medical recovery services (Carmen Oprea, PhD student; Prof. Petru Armean, MD, PhD); Rehabilitation in pulmonary diseases in Romania (Lecturer Elena Danteş, MD, PhD); Basic notions and news in photobiology and heliotherapy (Prof. Gelu Onose, MD, PhD); Chronic regional pain syndrome - a subdiagnosed pathology which interests many specialties (Recovery, Neurology, Cardiology, Orthopedy, Rheumatology, Plastic surgery) (Sibel Demirgian, MD, PhD); An overview of effective communication in medical life (Prof. Dumitru Borţun, PhD); The eyes and the sea – a relationship with high tide and low tide…(Assoc. Prof. Sanda Jurja, MD, PhD); Modulated therapy with radiofrequency in recovery medicine (Lecurer Dan Blendea, PhD); Sustainable development and natural resources: the challenge of e-innovation and quality of life in our society (Assoc. Prof. Zacharoula Andreopoulou, MD, PhD); Exploitation of natural resources management in health benefit Lake Techirghiol (Elena-Roxana Almăşan, PhD student); Idiopathic scoliosis – assessment and treatment (Prof. Elena Căciulan, MD, PhD, Phisiokinetotherapist; Nicoleta Daniela Calotă, MD, PhD, Kinesiotherapist); Rehabilitation of the low back pain syndrom through specific physical therapy techniques (Daniela Stanca, MD, PhD, Kinesiotherapist; Diana Voican, MD, PhD); Mechatronic wearable exoskeletons for bionic bipedal standing and walk assistance: a new synthetic approach (Prof. Gelu Onose, MD, PhD, Phisiokinetotherapist; Vladimir Cârdei; Ştefan T. Crăciunoiu; Valeriu Avramescu; Mikhail A. Lebedev, MD, PhD; Prof. Ioan Opris, MD, PhD; Prof. Marian Vladimir Constantinescu, MD, PhD), etc.

**Fig. 3:**
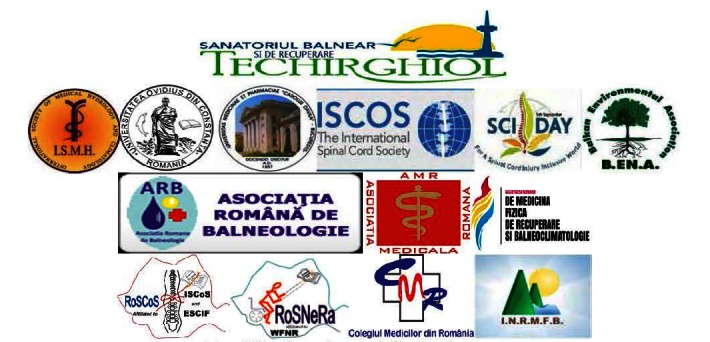
Media partners of the Conference

The event was advertised with the help of the following mass-media partners: National Tourism Authority, Techirgiol City Hall, College of Physicians in Romania – Constanta – and the Order of Nurses, Midwives and Medical Assistants in Romania – from Constanta branch.

**Executive Editor** **Professor Eng. Victor Lorin Purcarea, PhD**

